# Does Repeated Measurement of a 6-Min Walk Test Contribute to Risk Prediction in Children with Dilated Cardiomyopathy?

**DOI:** 10.1007/s00246-019-02244-7

**Published:** 2019-11-12

**Authors:** Marijke H. van der Meulen, Susanna den Boer, Gideon J. du Marchie Sarvaas, Nico A. Blom, Arend D. J. ten Harkel, Hans M. P. J. Breur, Lukas A. J. Rammeloo, Ronald Tanke, Willem A. Helbing, Eric Boersma, Michiel Dalinghaus

**Affiliations:** 1 Department of Pediatric Cardiology, Erasmus MC, University Medical Center Rotterdam, Rotterdam, The Netherlands; 2 Department of Pediatric Cardiology, University Medical Center Groningen, University of Groningen, Groningen, The Netherlands; 3 Department of Pediatric Cardiology, Leiden University Medical Center, Leiden, The Netherlands; 4 Department of Pediatric Cardiology, Academic Medical Center, Amsterdam, The Netherlands; 5 Department of Pediatric Cardiology, Wilhelmina Children’s Hospital, University Medical Center Utrecht, Utrecht, The Netherlands; 6 Department of Pediatric Cardiology, Free University Medical Center, Amsterdam, The Netherlands; 7 Department of Pediatric Cardiology, Radboud University Medical Center, Nijmegen, The Netherlands; 8 Department of Biostatistics, Erasmus MC, University Medical Center, Rotterdam, The Netherlands; 9 Department of Pediatric Cardiology, Erasmus University Medical Center, Dr. Molewaterplein 60, P.O. Box 2060, 3000 CB Rotterdam, The Netherlands

**Keywords:** Dilated cardiomyopathy, Heart failure, Pediatric cardiology, Risk factors, 6MWT

## Abstract

A single 6-min walk test (6MWT) can be used to identify children with dilated cardiomyopathy (DCM) with a high risk of death or heart transplantation. To determine if repeated 6MWT has added value in addition to a single 6MWT in predicting death or heart transplantation in children with DCM. Prospective multicenter cohort study including ambulatory DCM patients ≥ 6 years. A 6MWT was performed 1 to 4 times per year. The distance walked was expressed as percentage of predicted (6MWD%). We compared the temporal evolution of 6MWD% in patients with and without the study endpoint (SE: all-cause death or heart transplantation), using a linear mixed effects model. In 57 patients, we obtained a median of 4 (IQR 2–6) 6MWTs per patient during a median of 3.0 years of observation (IQR 1.5–5.1). Fourteen patients reached a SE (3 deaths, 11 heart transplantations). At any time during follow-up, the average estimate of 6MWD% was significantly lower in patients with a SE compared to patients without a SE. In both patients groups, 6MWD% remained constant over time. An absolute 1% lower 6MWD% was associated with an 11% higher risk (hazard) of the SE (HR 0.90, 95% CI 0.86–0.95 *p* < 0.001). Children with DCM who died or underwent heart transplantation had systematically reduced 6MWD%. The performance of all patients was stable over time, so repeated measurement of 6MWT within this time frame had little added value over a single test.

## Introduction

The 6-min walk test (6MWT) is a safe, simple, and well-accepted prognostic tool in adults with heart failure [1]. The test is used as an outcome measure in clinical trials, and short-term change in 6MWT is considered an indicator of prognosis as well [2–4].

In children, the 6MWT is also feasible and has been shown to be reproducible [5]. The distance walked in a 6MWT can be expressed as a percentage of predicted, taken into account height, gender, and age (6MWD%) [6]. The 6MWT has been successfully used to predict outcome in pediatric patients with pulmonary hypertension [7] and to evaluate therapy effects in patients with Duchenne muscular dystrophy [8] and pulmonary hypertension [9]. Previously, we showed that in children with dilated cardiomyopathy (DCM), a single 6MWD% below 63% identified patients with the highest risk of dying or heart transplantation (HTx) [10].

In this study, we report the results of repeated 6MWT in children with DCM. We studied the evolution of 6MWD% over time in relation to death or HTx. We aim to determine whether 6MWD% changes over time, and if so, if this change is associated with the risk of dying or HTx. We hypothesize that repeated measurement of 6MWT has added value over a single measurement in predicting these clinical outcomes.

## Methods

Data were collected in a multicenter, prospective study design. In total, patients from eight tertiary cardiac centers were included, covering potentially the whole Dutch population of children with DCM. Our research program started in October 2010 and ended in July 2017. In this period, we enrolled children with a previous diagnosis of DCM until 2010, relatively late after diagnosis, or with a new diagnosis from 2010 and onward, early after diagnosis. DCM was defined as the presence of impaired systolic function (fractional shortening (FS) ≤ 25%) and left ventricular (LV) dilation (LV end-diastolic dimension (LVEDD) >  + 2 *Z* score for body surface area). Patients with neuromuscular disease, cognitive impairment, or structural heart disease were excluded. The research program was organized in such a way that study visits coincided with routine outpatient clinic visits. In the first year after diagnosis, 6MWTs were obtained 1 to 4 times per year, and 1 to 2 times per year thereafter, dependent on the frequency of visits. This study was approved by the medical ethical committee of the Erasmus MC (MEC 2014–062) and performed in accordance with the declaration of Helsinki. All parents and children ≥ 12 years gave their written informed consent.

All included patients were asked to perform 6MWTs as part of our follow-up study program, at the outpatient clinic in the participating centers. The 6MWT was conducted according to the guidelines of the American Thoracic Society [11]. The local conditions at the outpatient clinics required adaptation of the walking track, as described previously [10]. In summary, patients were instructed to walk as far as possible on an 8-m track within a 6-min time frame. Patients were instructed and encouraged in a standardized manner. Running was not allowed, if necessary, patients could slow down or stop walking. After 6 min, the total amount of 16 m ‘laps’ was counted and the distance walked in a partially finished lap was added, which resulted in a total walking distance (6 MWD). If the patient stopped walking before the end of 6 min, e.g., due to fatigue, we accepted the walked distance as 6MWD. All 6MWT study data were collected by study personnel and stored in the study database, the treating cardiologists were blinded to the study results. The distance covered was expressed as percentage of predicted (6MWD%) according to Geiger et al., accounting for height, gender, and age [6]. Calculation of 6MWD% was done after closure of the database and used for study purposes only.

As part of the follow-up study, in addition to the 6MWT, multiple data were recorded on the same patient visit: weight and height, current heart failure medication and dosage, New York Pediatric Heart Failure Index (NYPHFI), NT pro-BNP, and a standardized echocardiogram including left ventricular end-diastolic dimension (LVEDD) and fractional shortening (FS).

The study endpoint was death or heart transplantation (HTx). The decision to list a patient for HTx was made by a team of treating physicians based on commonly accepted criteria [12]. The team was blinded to the results of the 6MWT. In addition, we recorded the status of the patients at the end of the study, after their last study visit: ongoing disease or recovered. Recovery was defined as 2 consecutive echocardiograms with normalized LVEDD and FS, the date of the first normalized echocardiogram was considered as date of recovery. Echocardiograms were analyzed and reviewed by study personnel who were blinded to the patient’s name, previous echocardiograms, and other study results.

Continuous variables with normal distribution are described as mean (standard deviation, SD), or as median (interquartile range, IQR) otherwise. Categorical variables are described as numbers and percentages. Differences in 6MWT characteristics between patients with and without the study endpoint were compared by Student’s *t* tests (normal distribution) or Mann–Whitney tests.

We applied a linear mixed effects model (LMEM) for longitudinal data to study the temporal evolution of 6MWD%, while accounting for the correlation between measurements in the individual patient. Subsequently, the LMEM was combined with a Cox proportional hazard regression model in a so-called joint model (JM) to explore the association between 6MWD% (repeatedly measured) and the study endpoint. We included time since diagnosis as covariate in the JM.

We studied cumulative event-free survival of the study endpoint by the method of Kaplan–Meier. In a previous study, we showed that pediatric DCM patients with baseline 6MWD% < 63% have high risk of death or heart transplant during prolonged follow-up [10]. Against this background, we separated our study cohort according to this threshold, and compared cumulative event-free survival by a log-rank test.

The level of statistical significance for all analyses was set at *p* = 0.05. Analyses were performed using IBM SPSS statistics 24 (IBM, New York, USA) and R statistical software version 3.5.1 (package JMbayes).

## Results

**Fig. 1:**
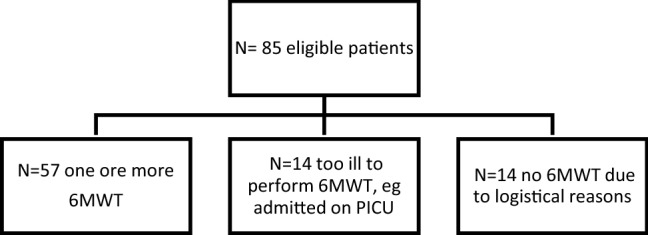
Flow diagram of eligible patients and included patients

Eighty-five patients met the inclusion criteria, of which 57 performed at least one 6MWT. Twenty-eight patients did not perform a 6MWT: 14 patients were too ill (e.g., admitted at the PICU) and reached an endpoint before a 6MWT could be obtained. Another 14 patients did not perform a 6MWT due to logistical reasons (Fig. 1). Nineteen of the 57 patients were included early after diagnosis, whereas 38 patients had been diagnosed before 2010 and were included relatively late after diagnosis. Patient characteristics are described in Table 1. At the first 6MWT, the median age was 11.1 years (IQR 7.3–14.5), median time since diagnosis was 3.6 years (IQR 0.5–7.1), and median time since study inclusion was 0.1 years (IQR 0.0–0.9). Idiopathic DCM was diagnosed in 47%, and the majority of patients were treated with ACE- inhibitors (90%) and β-blockers (78%). The median NYPHFI at the baseline 6MWT was 8 (IQR 4–11; Table 1). For 49 patients, we previously reported the results of a single 6MWT and outcome [10].

**Table 1:** Characteristics of the study cohort (*n* = 57) at the moment of baseline 6-min walk test

Time since diagnosis, years, median (IQR)	3.5 (0.5–7.1)
Time since study inclusion, years, median (IQR)	0.1 (0.1–0.9)
Gender, female, *n* (%)	68 (47%)
Age, years, median IQR	11.1 (IQR 7.3–14.5)
Cause of DCM (*n*, %)	
Idiopathic	26 (47)
Genetic or familial	8 (14)
Other	23 (40)
Heart failure score, NYPHFI median (IQR)	8 (4–11)
Medication, *n* (%)	
β-blockers	45 (78%)
Ace inhibitors	51 (90%)
Anti-coagulants	29 (51%)
Diuretics	32 (56%)
Echocardiografic parameters, mean (SD)	
LVEDD *z* score	4.7 (2.9)
SF	18.3 (6.7)
NT-pro BNP, pg/ml, median (IQR)	1873 (390–5021)
Endpoint	
Death/transplantation	14
Ongoing disease	39
Recovered	4
Number of 6MWT per patient, median (IQR)	4 (2–6)
6MWT, meters, mean (SD)	462 (122)
6MWD% of predicted, %, mean (SD)	69 (17)
Total follow-up time per patient, years, median (IQR)	3.0 (1.5–5.1)

*DCM* dilated cardiomyopathy, *6MWT* 6-min walk test, *NYPHF* New York University Pediatric Heart Failure Index, range 0–30, *LVEDD* Left ventricular end-diastolic dimension, *SF* shortening fraction

Median observation time per patient was 3.0 years (IQR 1.5–5.1), in patients with the study endpoint, median observation was 1.3 years (IQR 0.2–2.1), and in patients without the study endpoint, median observation was 3.8 years (IQR 2.1–5.3; *p* = 0.001). In this time frame, 277 6MWTs were performed, and 47 of the 57 patients performed more than one 6MWT. The median number of 6MWTs per patient was 4 (IQR 2–6). The median number of 6MWTs per year follow-up was 2.7 (IQR 1.0–2.9). The mean distance walked, including all available tests, was 462 m (± 122), the mean 6MWD% was 69% (± 17) (Table 1).

In the course of the study, 14 of the 57 patients in whom a 6MWT was available, reached a study endpoint: 11 patients were transplanted at a median of 5.7 years after diagnosis (IQR 2.5–11.0) and 3 patients died, 1.3, 2.3, and 8.3 years after diagnosis. Death was caused by DCM-related ventricular arrhythmia in one patient, end-stage heart failure in a setting of a contra-indication for HTx in the second patient, and multi-organ failure in a patient with an additional glycogen storage disease.

In patients who reached a study endpoint, median time from the last 6MWT to the study endpoint was 0.25 years (IQR 0.16–0.77). Median time since diagnosis to the first 6MWT was 3.2 years (IQR 0.4–6.8), which was the same as in patients who did not reach a study endpoint. The median number of 6MWTs was the same in patients with and without a study endpoint (Table 2). At the last follow-up visit, 4 of the remaining 43 patients had recovered, whereas 39 patients had ongoing disease. Three recovered children showed an increase in 6MWD%, the fourth performed only one test. The low number of recovered patients did not allow for statistical analysis.

**Table 2:** Repeated measurement of 6MWT, comparing patients with a study endpoint to patients without a study endpoint

	All patients (*n* = 57)	SE (*n* = 14)	No SE (*n* = 43)	*p* value
Time Dx-fist 6MWT^a^	3.6 (0.5–7.1)	3.2 (0.4–6.8)	3.6 (0.5–7.4)	0.81
Time last 6MWD% to PEP or end of study^a^	0.08 (0.0–0.46)	0.25 (0.16–0.77)	0.00 (0.00–0.38)	0.004
Total FU time^a^	3.0 (1.3–5.1)	1.3 (0.2–2.1)	3.8 (2.1–5.3)	0.007
Number of 6MWT per patient	4 (2–6)	3 (1–6)	4 (2–7)	0.70
Number of 6MWT per year follow-up	2.7 (1.0–2.9)	3.7 (2.4–6.6)	1.6 (0.9–2.5)	0.004

Comparison of patients with SE vs patients without SE using Mann–Whitney test

All numbers are median and Inter Quartile Range (IQR)

*SE* study endpoint, *Dx* diagnosis

^a^Years

**Fig. 2:**
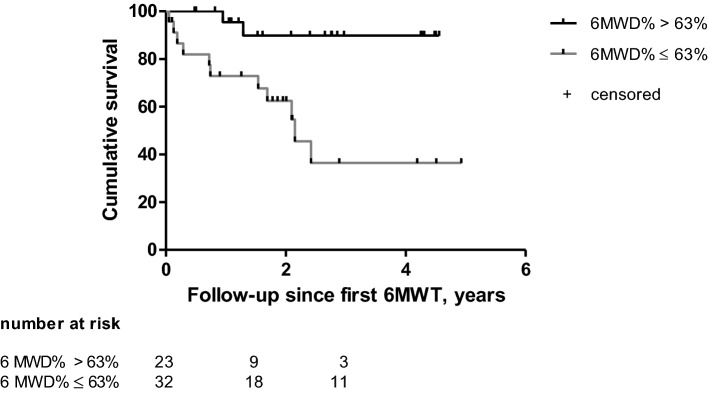
Transplant-free survival curves of DCM patients with 6MWD ≤ 63% of predicted compared to patients with 6MWD > 63% of predicted

At the first 6MWT, median 6MWD% was 68% (IQR 53–82%). In patients with a study endpoint, 6MWD% was 53% (IQR 33–61), compared to 74% (IQR 60–84) in patients without a study endpoint (*p* = 0.003) (Table 2). Transplant-free survival was significantly higher in patients with a first 6MWD% ≥ 63%. In children with a first 6MWD% ≥ 63%, 1-year transplant-free survival was 96% (95% CI 89 to 100), 2-year transplant-free survival was 92% (95% CI 81 to 100) in contrast to children with a first 6MWD% < 63% in whom 1-year transplant-free survival was 74% (95% CI 56 to 92) and 2-year transplant-free survival was 64% (95% CI 44 to 84) (log-rank test *p* = 0.002; Fig. 2). Thus, a 6MWD% lower than 63% was associated with an increased risk of heart transplantation or death (hazard ratio 10.8; 95% CI 2.4 to 49).

**Fig. 3:**
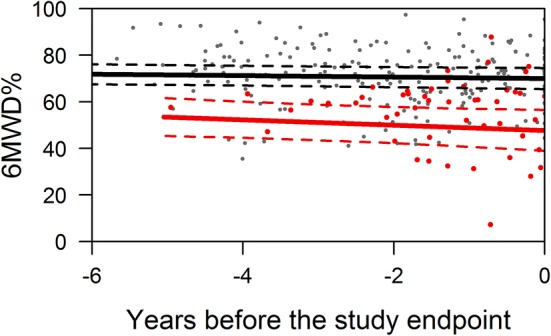
Serial measurement of 6MWD expressed as percentage of predicted, time before study endpoint. The average estimates of the longitudinal trajectory of 6MWD%: the black line indicates the patients without a study endpoint, the red line the patients with a study endpoint. The dashed lines depict the 95% confidence interval

**Fig. 4:**
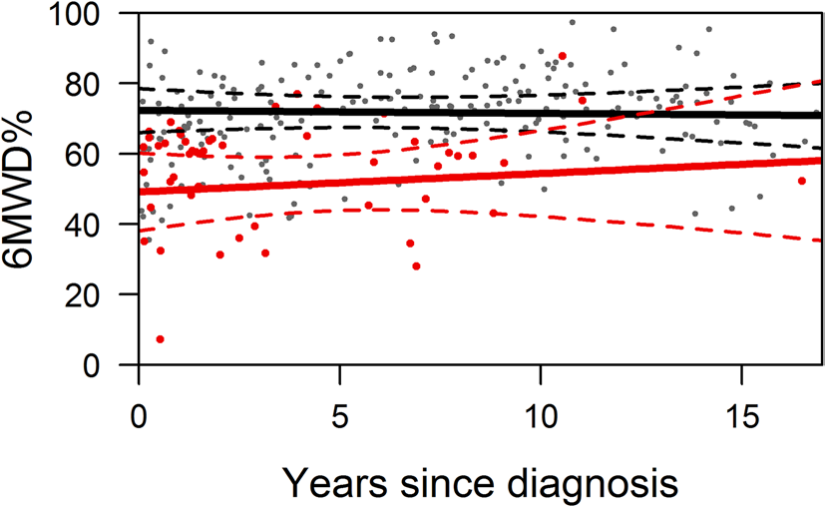
Serial measurement of 6MWD expressed as percentage of predicted, time since diagnosis. The average estimates of the longitudinal trajectory of 6MWD%: the black line indicates the patients without a study endpoint, the red line the patients with a study endpoint. The dashed lines depict the 95% confidence interval

**Fig. 5:**
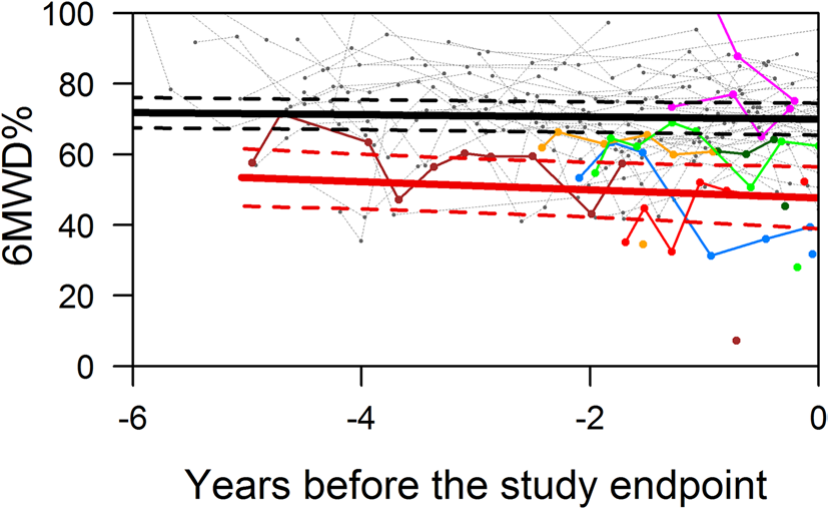
Serial measurement of 6MWD expressed as percentage of predicted, time before study endpoint. The average estimates of the longitudinal trajectory of 6MWD%: the black line indicates the patients without a study endpoint, the red line the patients with a study endpoint. The dashed lines depict the 95% confidence interval. The individual patients are plotted, the colored lines indicate the patients with the study endpoint

At any time during follow-up, the average estimate of 6MWD% was significantly lower in patients with a study endpoint compared to patients without a study endpoint (Fig. 3). Furthermore, we found no meaningful change in 6MWD% over time both in patients with and without a study endpoint (Fig. 3). Notably, in patients who reached an endpoint, 6MWD% did not change over time and did not suddenly decrease prior to the endpoint. When we plotted the results of the 6MWD% against years since diagnosis, we also found that they were constant over time (Fig. 4), indicating that there was no systematic difference in the results obtained early and later after diagnosis. The variability of the 6MWD% within the individual patient was considerable (Fig. 5), suggesting that obtaining more than one 6MWT would be useful to obtain a reliable mean estimate of the patient. An absolute 1% lower 6MWD% was associated with a 11% higher risk (hazard) of the study endpoint (HR 0.90, 95% CI 0.86–0.95 *p* < 0.001). In clinical practice this means that when comparing 2 patients at any given moment after diagnosis, the one with a 1% lower 6MDW% has an 11% increased risk of death or HTx.

## Discussion

In this study, we confirm the usefulness of the 6MWT as a tool to identify children with DCM at a high risk of adverse outcome. In the studied time frame, 6MWD% was significantly lower in patients who reached the study endpoint of death or HTx, compared to those who did not. The 6MWD% remained constant throughout 3 years of observation, both in patients who reached the study endpoint, and in those who did not. As 6MWD% tends to vary within the individual patient, at least two 6MWTs seem to be needed to obtain a reliable adequate indication of 6MWD%. Thereafter, repeating the 6MWT seems to have little added value.

In adult heart failure literature, data on the use of serial 6MWT are scarce, but a number of studies have shown that repeating 6MWT is useful to evaluate effectiveness of therapeutic interventions. A short-term improvement in 6 MWD after a drug intervention in hospitalized patients with chronic heart failure was a significant independent predictor of survival [2]. Likewise, in patients with moderate to severe heart failure, cardiac resynchronization therapy results in significant improvement in 6MWD and in survival [3]. In contrast, in the majority of trials that were reviewed, the 6MWD did not increase after a pharmacological intervention in patients with chronic stable heart failure [13]. Combining both findings would indicate that repeating the 6MWT is helpful to evaluate the effect of a therapeutic intervention on survival in patients with moderate to severe heart failure.

Upfront, we expected that 6MWD% would decrease in the patients who reached an endpoint. Also, we hypothesized that 6MWD% might increase in the combined group of patients who recover or have ongoing disease. However, our results did not confirm these expectations as 6MWD% was stable from early after diagnosis onward, both in children who reached an endpoint and those who did not. Moreover, it was significantly lower in those reaching an endpoint throughout the time of observation (Fig. 3). Notably, 6MWTs were only obtained in patients who were relatively stable. In two potentially unstable phases of the disease, directly after diagnosis and shortly before death or HTx, a substantial number of patients did not perform a 6MWT. Sixteen percent of potentially eligible patients never performed a 6MWT, because they were too ill and reached an endpoint before a 6MWT could be done. In that respect, not being able to perform a 6MWT can be considered an ominous sign. On the other end of the spectrum, most patients who did perform one or more 6MWT and reached an endpoint within the study period did not perform a 6 MWT in the last phase of the disease: 8 of the 14 children with a study endpoint were listed for HTx after the last available 6MWT, and were hospitalized awaiting transplantation.

There are several limitations to this study. Firstly, we could not obtain a 6MWT in a number of patients due to logistical difficulties that come with a multicenter study. However, the 14 patients we missed were not significantly different from the study cohort in terms of age, gender, cause of DCM, NYPHFI, and percentage of study endpoints (results not shown). We believe therefore that the study cohort was an unbiased selection and that missing 6MWTs have not led to an important change in the results and conclusions of our study. Secondly, the studied cohort is relatively small, though compared to other pediatric studies using 6MWT to predict outcome comparable or larger in the number of children that we studied and the number of 6MWTs that were obtained. REFS Moreover, it is the first study on serial measurements of 6MWD% in children with DCM. Nevertheless, one could hypothesize that in a study with a larger cohort of children with DCM patients, we would have been able to demonstrate that 6MWD% significantly changes over time and that this change would identify patients with a higher risk of reaching an endpoint. The graph in Fig. 3, however, does not provide much support for that idea as it depicts that there is no change at all over time in 6MWD%, in both groups. We pose that expanding the study cohort would not lead to a fundamental change in the results we found. Thirdly, the great majority of study endpoints reached in this study was HTx, the number of deaths was low. This is in line with other studies reporting on outcomes in children with DCM, showing that in general, deaths occur relatively early after diagnosis and that HTx takes place later after diagnosis [14–16]. In this respect, it is fair to state that 6MWTs identify the patients at a high risk for HTx rather than the patients at a high risk to die. Fourthly, a similar amount of 6MWTs were performed in children who did and did not reach a study endpoint, but the 6MWT were performed in a shorter time frame. This reflects the clinical practice in which the most seriously affected children are seen more often at the outpatient clinic. Lastly, the patient cohort we studied contains a low number of children that recover compared to our previous reports and large cohorts of children with DCM. The fact that children under 6 years were not included in this study is probably an important explanation as recovery in this age group is relatively common [14–17].

We conclude that, based on the current study, 6MWT is a useful tool to identify children with DCM at a high risk of death or heart transplantation. In children who are able to perform a 6MWT, 6MWD% remains constant over time, early after diagnosis and in the years thereafter, in those reaching an endpoint, 6MWD% is significantly lower throughout time, than in those not reaching an and endpoint. Initially, at least 2 6MWT are needed to reliably estimate 6MWD%, thereafter repeating 6 MWT has little added value.
